# Optimization of Protoplast Isolation from Leaf Mesophylls of Chinese Cabbage (*Brassica rapa* ssp. *pekinensis*) and Subsequent Transfection with a Binary Vector

**DOI:** 10.3390/plants10122636

**Published:** 2021-11-30

**Authors:** Ganeshan Sivanandhan, Solhee Bae, Chaemin Sung, Su-Ryun Choi, Geung-Joo Lee, Yong-Pyo Lim

**Affiliations:** 1 Molecular Genetics and Genomics Laboratory, Department of Horticulture, College of Agriculture and Life Science, Chungnam National University, Daejeon 34134, Korea; gsivacnu@gmail.com (G.S.); bsh3764@naver.com (S.B.); agridays8@gmail.com (C.S.); srchoi@cnu.ac.kr (S.-R.C.); 2 Department of Horticulture, College of Agriculture and Life Science, Chungnam National University, Daejeon 34134, Korea; gjlee@cnu.ac.kr; 3 Department of Smart Agriculture Systems, College of Agriculture and Life Science, Chungnam National University, Daejeon 34134, Korea

**Keywords:** green fluorescent protein, PEG-mediated transfection, protoplast yield, transfection efficiency, transient gene expression

## Abstract

Chinese cabbage is an important dietary source of numerous phytochemicals, including glucosinolates and anthocyanins. The selection and development of elite Chinese cabbage cultivars with favorable traits is hindered by a long breeding cycle, a complex genome structure, and the lack of an efficient plant transformation protocol. Thus, a protoplast transfection-based transformation method may be useful for cell-based breeding and functional studies involving Chinese cabbage plants. In this study, we established an effective method for isolating Chinese cabbage protoplasts, which were then transfected with the pCAMBIA1303 binary vector according to an optimized PEG-based method. More specifically, protoplasts were isolated following a 4 h incubation in a solution comprising 1.5% (*v*/*v*) cellulase, 0.25% (*v*/*v*) macerozyme, 0.25% (*v*/*v*) pectinase, 0.5 M mannitol, 15 mM CaCl_2_, 25 mM KCl, 0.1% BSA, and 20 mM MES buffer, pH 5.7. This method generated 7.1 × 10^6^ protoplasts, 78% of which were viable. The *gfp* reporter gene in pCAMBIA1303 was used to determine the transfection efficiency. The Chinese cabbage protoplast transfection rate was highest (68%) when protoplasts were transfected with the 40 µg binary vector for 30 min in a solution containing 40% PEG. The presence of *gusA* and *hptII* in the protoplasts was confirmed by PCR. The methods developed in this study would be useful for DNA-free genome editing as well as functional and molecular investigations of Chinese cabbage.

## 1. Introduction

Chinese cabbage (*Brassica rapa* ssp. *pekinensis*) is a major agro-economic leafy vegetable crop worldwide. It is an excellent source of numerous phytochemicals (e.g., glucosinolates, phenolics, flavonoids, anthocyanins, and carotenoids) that are potentially useful because of their nutritional value and medicinal properties [1]. Hence, the estimated global production of cabbage has increased from 65.58 megatons in 2009 to 70.15 megatons in 2019 [2]. Although the consumer demand for Chinese cabbage keeps increasing, the production of this crop has been constrained by various biotic and abiotic factors [3]. Moreover, the associated methods are time-consuming and laborious. Alternatively, some agronomically important traits have been incorporated into the Chinese cabbage genome via *Agrobacterium*-mediated transformation, but this approach may lead to undesirable genomic mutations [3].

Because of the global demand for Chinese cabbage, researchers have been actively trying to enhance its agronomic traits using various biotechnological tools. Genome editing through CRISPR/Cas9-mediated modifications has recently attracted attention regarding its utility for introducing or improving several critical traits in agriculturally valuable crops. In particular, protoplast-based ribonucleoprotein delivery method turned out to be more advantageous for editing in the desired gene than *Agrobacterium*-mediated transformation in terms of lower off-target mutant production [4]. Lin et al. [5] reported transient protoplast transfection as one of the best methods to check the efficacies of multiple mutagenesis and silencing studies. The isolation and transfection of protoplasts have been reported for numerous agriculturally important crops, including rice [6], soybeans [7] and sugarcane [8]. Some studies on protoplast isolation have been conducted on *B. oleracea*, *B. rapa* L. ssp. *pekinensis*, *B. juncea*, *B. sinensis* [9,10,11,12,13,14,15,16]; however, these were not standardized and investigated in greater detail. Sindhu and Cohen [9] reported on the subcellular localization of spermidine synthase in a protoplast of *B. pekinensis* by adjusting their concentration to 1–2 × 10^6^ per ml. Balint and Cohen [10,11] achieved turnip yellow mosaic virus replication in a protoplast of *B. pekinensis* by adjusting their concentration to 3–4 × 10^5^ per ml. Similarly, Boyer et al. [12] described turnip yellow mosaic virus replication in protoplasts of *B. napus* or *B. sinensis* by adjusting their concentration to 1.5 × 10^6^. Liu et al. [13] performed protoplast fusion between *B. oleracea* and *B. rapa* L. ssp. *pekinensis* by adjusting their concentration to 1 × 10^5^ per ml. Lian et al. [14] adjusted protoplast concentration to 5 × 10^5^ per ml for the production of somatic hybrids from rapid cycling *B. rapa* and *B. juncea* by protoplast fusion. Murovec et al. [15] adjusted the protoplast concentration to 5 × 10^5^ for RNP complex delivery into *B. rapa* L. ssp. *pekinensis* and *B. napus* protoplasts. Jeong et al. [16] optimized the protoplast concentration of 2 × 10^5^ for RNP complex delivery into *B. rapa* L. ssp. *pekinensis* protoplasts. 

Yield, viability and transformation efficiency are important factors for protoplast isolation, culture and transfection in plants. These factors widely determine the further studies on CRISPR/Cas9, protein–protein interaction, protein localization, function and the regulation of genes [5]. The objective of this study was to develop an optimal method for isolating Chinese cabbage protoplasts from mesophyll cells and transfecting them with a large (approximately 12 kb) vector (pCAMBIA1303). Specifically, we optimized several factors influencing protoplast yield and viability, including mannitol concentration, cell wall-digesting enzyme concentration, incubation time, polyethylene glycol (PEG) concentration, vector concentration, and the incubation time for the transfection. The integration of *gusA* and *hptII* genes in the transfected protoplasts was confirmed by PCR.

## 2. Results

### 2.1. Optimization of Factors Influencing Protoplast Yield

**Figure 1:**
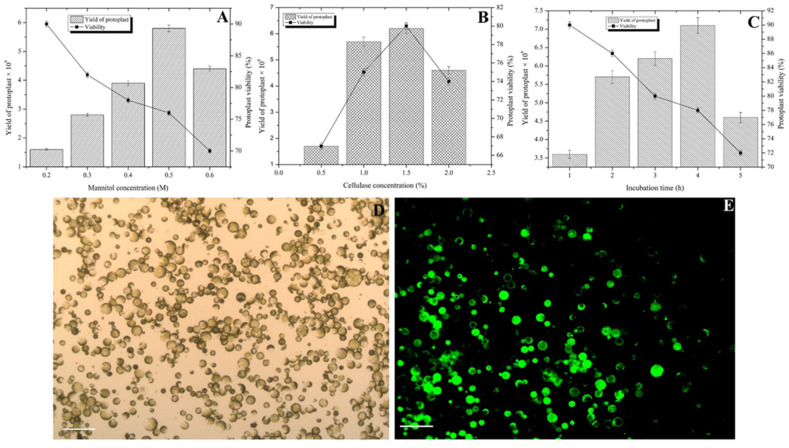
Standardization of factors that influence the yield and viability of protoplasts. Data obtained from different concentrations of mannitol (**A**), cellulase (**B**), various incubation time (**C**). (**D**) Light microscopic view of isolated protoplasts from leaf mesophyll tissues of *B. rapa* in W5 solution. (**E**) Ultraviolet microscopic view of isolated protoplasts from leaf mesophyll tissues of *B. rapa* stained with fluorescein diacetate (30 min incubation). Results obtained from an optimized enzymatic mixture that consisted of cellulase (1.5%) with a combination of macerozyme at 0.25%, pectinase at 0.25%, 0.5 M of mannitol, 15 mM CaCl_2_, 25 mM KCI, 0.1% BSA, and 20 mM MES buffer (pH 5.7) with 4 h incubation time at 50 rpm rotation at 25 °C. Data represent mean ± SE of three replicates. Mean separations were performed using Duncan’s multiple range test, and significance was determined at the 5% level. *Bar*~100 µm.

Various concentrations of mannitol were tested to determine the optimum concentration to maximize the recovery of viable protoplasts. Increasing the mannitol concentration up to 0.5 M increased the protoplast yield and viability. At 0.5 M mannitol, 5.8 × 10^6^ protoplasts per g FW were recovered, 77% of which were viable (Figure 1A). Increasing the mannitol concentration beyond 0.5 M decreased the protoplast yield and viability (Figure 1A). Hence, 0.5 M mannitol was suitable for the efficient isolation of Chinese cabbage protoplasts.

The use of different cellulase concentrations in the digestion mixture notably influenced Chinese cabbage protoplast yield and viability. Of the tested concentrations, 1.5% cellulase was most effective, as it produced 6.2 × 10^6^ protoplasts per g FW, 80% of which were viable (Figure 1B). Further increasing the cellulase concentration to 2% adversely affected protoplast yield and viability. Lower cellulase concentrations (0.5% and 1.0%) resulted in a poor yield, but they positively affected protoplast viability (Figure 1B). Thus, 1.5% cellulase produced optimal protoplast yield and viability.

After optimizing the mannitol and cellulase concentrations, the effect of incubation time was examined in a shaker at 25 °C. Figure 1C indicates that 4 h incubation time produced highest yield, 7.1 × 10^6^ protoplasts per g FW, 78% of which were viable (Figure 1D,E). Increasing the incubation time from 1 to 4 h increased the yield from 3.6 × 10^6^ protoplasts to 7.1 × 10^6^ protoplasts per g FW, but it decreased protoplast viability from 90% to 78%. Accordingly, Chinese cabbage protoplasts were efficiently isolated following a 4 h incubation in a solution containing 1.5% cellulase and 0.5 M mannitol (Figure 1D,E).

### 2.2. Optimization of Factors Influencing Transfection with a Binary Vector

**Figure 2:**
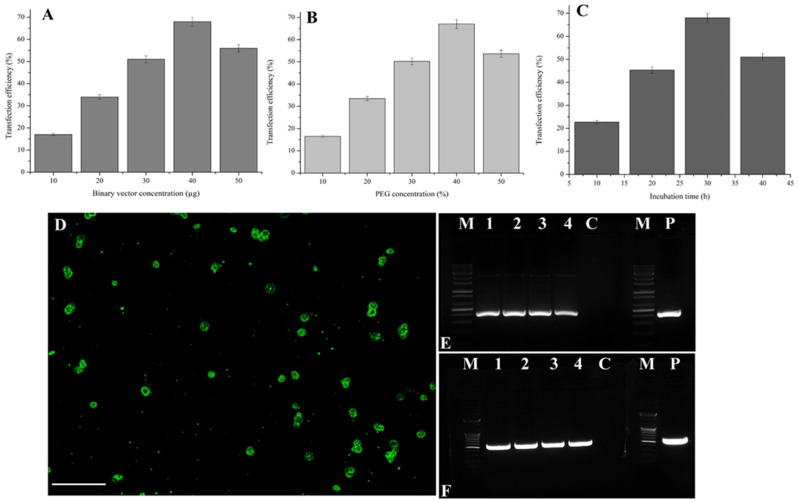
Standardization of factors that influence the transfection efficiency of a binary vector. Data obtained from different concentrations of binary vector, pCAMBIA1303 (**A**), PEG (**B**), and various incubation time (**C**). (**D**) Ultraviolet microscopic view of pCAMBIA1303 transfected-protoplasts isolated from leaf mesophyll tissues of *B. rapa*. PCR amplification of *hptII* (**E**), and *gusA* (**F**) from extracted DNA of pCAMIBA1303-transfected protoplasts. M: marker (100 bp plus), 1 to 4: transfected protoplasts, C: negative control, P: positive control. The result was made from the transfection of 40 µg of pCAMBIA1303 vector, 40% PEG, and 30 min transfection time followed by 48 h incubation under total darkness at 25 °C. Data represent mean ± SE of three replicates. The mean separations were performed using Duncan’s multiple range test, and significance was determined at the 5% level.

Transfection efficiency was evaluated using 2 × 10^6^ protoplasts and various concentrations of pCAMBIA1303. The transfection efficiency was 17% at 10 µg pCAMBIA1303 was added to the protoplasts (Figure 2A). However, the transfection efficiency was increased to 68% when the 40 µg binary vector was added to the protoplasts (Figure 2A). Further increases to the 50 µg binary vector decreased the transfection efficiency to 56%. Accordingly, transfecting 2 × 10^6^ protoplasts in a 100 µL solution with 40 µg binary vector was optimal.

After optimizing the concentration of binary vector, various PEG concentrations (10–50%) were tested. At 10% PEG, the transfection efficiency was 16.3%. Increasing the PEG concentration to 40% increased the transfection efficiency to 67.3% (Figure 2B). The effect of the incubation time (10–40 min) was assessed for the transfection of protoplasts in a solution containing 40% PEG and 40 µg binary vector (Figure 2C). The incubation time substantially influenced the protoplast transfection efficiency. Specifically, the transfection efficiency was 22.66% when the incubation time was 10 min, but it was increased to 68% at 30 min (Figure 2C,D). The pCAMBIA1303-transfected protoplast exhibited good GFP expression at a wavelength of 480 nm. Notably, there was a strong relationship among binary vector concentration, protoplast yield, and incubation time (Figure 2).

### 2.3. Confirmation of Vector Integration by PCR

Protoplasts transfected with pCAMBIA1303 (lanes 1 to 4) and the respective controls were analyzed by PCR using *gusA*- and *hptII*-specific primers. The expected amplified fragments (i.e., 515 bp for *gusA* and 407 bp for *hptII*) were detected for the protoplasts transfected with the binary vector (Figure 2E,F) confirming the integration of GUS and hygromycin-resistance genes in the transfected protoplasts. Amplified fragments were undetectable for the controls.

## 3. Discussion

The CRISPR/Cas9 system has been successfully used to generate elite plant varieties exhibiting modified traits. Most of the earlier related studies used protoplasts to analyze and select ideal sgRNAs for specific tasks (e.g., gain or loss of function). Thus, higher yields of high-quality protoplasts are required for genome editing research. Chinese cabbage protoplasts were recently transfected with Cas9-gRNA ribonucleoprotein [16,17]. However, earlier studies did not optimize the major factors affecting protoplast isolation and transfection with a binary vector (e.g., pCAMBIA1303). Therefore, the results of the current study may be more useful for the isolation and transfection of Chinese cabbage protoplasts from leaf mesophylls using CRISPR/Cas9 binary vectors or ribonucleoprotein.

Plasmolysis during protoplast isolation is affected by the type and concentration of the osmoticum, which helps to maintain the turgor pressure in the resulting protoplasts [18]. In this study, the Chinese cabbage protoplasts’ yield (5.8 × 10^6^) and viability (77%) were high when 0.5 M mannitol was used (Figure 1A). Protoplast yield and viability increased significantly when mannitol concentration was increased from 0.2 to 0.5 M, but further increases decreased both the yield and viability of protoplasts. Moreover, protoplast aggregation was observed at 0.6 M mannitol. In contrast, at low mannitol concentrations (i.e., 0.2 and 0.3 M), the protoplasts were unstable and ruptured easily (data not shown). These observations may be explained as follows: first, the increase in the entry of water molecules because of the lack of a cell wall may cause the protoplasts to rupture. Second, protoplasts may shrink in hypertonic solutions as water molecules diffuse out because of osmosis. This may lead to the aggregation of protoplasts, which was previously observed by Kang et al. [19] in a study on *Chrysanthemum* protoplasts. Huo et al. [18] used 0.5 M mannitol to isolate protoplasts derived from *Liriodendron* hybrid plants.

Of the tested cellulase concentrations, 1.5% cellulase produced the highest protoplast yield, followed by 2% and 1% cellulase (Figure 1B). Higher concentrations were likely unnecessary because of the moderate deposition of cellulose in the Chinese cabbage cell wall. However, protoplast viability decreased as the cellulase concentration increased, which was consistent with the findings of an earlier study by Adedeji et al. [20] involving *Chrysanthemum* protoplast. High enzyme concentrations may have detrimental effects on plasma membrane integrity and the physiological and metabolic activities of protoplasts [21]. Most of the previous related studies used cellulase in two types of enzyme mixtures [20,22]. Cellulase at concentrations between 0.3% and 2% have been used to obtain high protoplast yields for diverse plants [8,19]. Moreover, explant type and age also influence the yield and viability of plant protoplasts [23]. Plant cell wall degradation by the regulated activities of specific enzymes is an extremely complex process that is mediated by transcription factors and regulators, including ACE1, ACE2, XYR1, ACE3, VIB1, and the HAP complex [24].

Yoo et al. [25] confirmed that the ideal incubation time for cell wall digestion depends on various factors, including the source material, the study objectives, the desired responses, and the chemicals used for isolating *Arabidopsis thaliana* protoplasts. An incubation time of 1 or 2 h resulted in relatively few protoplasts because of the insufficient enzyme activity for cell wall degradation. In contrast, increasing the incubation time (≥5 h) increased the protoplast yield as well as the amount of cell debris, but decreased protoplast viability in this study. In the current study, a 4 h incubation was considered optimal because it resulted in a high protoplast yield (7.1 × 10^6^) and moderate protoplast viability (76%) (Figure 1C–E). This was consistent with the findings of earlier studies on *A. thaliana* and cucumber protoplasts by Yoo et al. [25] and Huang et al. [23], respectively. In fact, the optimum digestion time varies depending on the enzymes and plant materials used. For example, Adedeji et al. [20] used a 4 h incubation to isolate protoplasts from *Chrysanthemum*, which was shorter than the 6 h incubation used by Kang et al. [19] to isolate protoplasts from *Petunia*. Sun et al. [26] produced protoplasts from Chinese kale during a 9 h incubation, whereas Wang et al. [8] suggested a 5 h incubation was appropriate for isolating sugarcane protoplasts. 

Sindhu and Cohen [9] reported 0.5% cellulase, 0.5% macerozyme and 0.4 M mannitol for the isolation of protoplasts from the leaves of *B. pekinensis* with 18 h incubation. Balint and Cohen [10,11] reported 2% cellulase R10, 0.1% pectolyase and Y-23 and 0.6 M mannitol for the isolation of protoplasts from the leaves of *B. pekinensis* with 3 h incubation. Liu et al. [13] reported 1% cellulase, 0.8% pectinase, 0.3% macerozyme R-10, 0.02% pectolyase Y-23 and 9% D-mannitol for the isolation of protoplasts from the microspores of *B. oleracea* and *B. rapa* L. ssp. *pekinensis* with incubation for 2 to 3 h. Lian et al. [14] reported 2% cellulysin, 0.5% macerozyme and 0.4 M mannitol for the isolation of protoplasts from the leaves of rapid cycling *B. rapa* with incubation for 16 to 20 h. Murovec et al. [15] reported 0.5% cellulase Onozuka RS, 0.1% pectolyase Y-23 and 0.4 M mannitol for the isolation of protoplasts from the leaves of *B. rapa* L. ssp. *pekinensis* and *B. napus* with incubation for 2.5 h. Jeong et al. [16] reported 1% cellulase R10, 0.5% macerozyme R10 and 0.45 M mannitol for the isolation of protoplasts from the seedlings of *B. rapa* L. ssp. *pekinensis* with 12 h incubation. In the present study, higher yield of protoplasts (7.1 × 10^6^) from leaf mesophylls was obtained using optimized factors such as 1.5% (*v*/*v*) cellulase, 0.25% (*v*/*v*) macerozyme, 0.25% (*v*/*v*) pectinase, 0.5 M mannitol at 4 h incubation.

The vector concentration is an important consideration for transfecting protoplasts for functional analyses. Regarding Chinese cabbage, the pCAMBIA1303 concentration of 40 µg resulted in the highest transfection rate (68%) under optimized conditions (Figure 2A). In earlier studies, the ideal vector concentration for transfecting protoplasts was determined for the following species: 30 µg for sugarcane, 20 µg for cucumber, and 10 µg for Chinese kale [8,23,26]. In the present study, the transfection rate increased significantly when the concentration of the binary vector was increased. However, most related studies indicated that vector size was a critical factor in influencing the success of protoplast transfection. A transfection rate of 45% was reported involving rice protoplasts using a large plasmid (approximately 12 kb) [27], which was higher than the transfection rate (22%) in another study involving switchgrass protoplasts transfected with a 16 kb vector [28]. However, *A. thaliana* protoplasts can be efficiently transfected (90%) by an approximately 4 kb vector [25]. In the present study, Chinese cabbage protoplasts were efficiently transfected (68%) by a large vector (approximately 12 kb). These results were possible because of the optimization of the factors influencing transfection.

Polyethylene glycol facilitates the transfection of protoplasts by vectors, but the effect of the PEG concentration is substantially influenced by other factors. The present study revealed that 40% PEG was optimal for maximum transfection (Figure 2B). Protoplast transfection was hindered at PEG concentrations exceeding 40%, which led to protoplast aggregation and low protoplast viability. Similar results were obtained in studies by Yao et al. [29] and Wang et al. [8] on sweet cherry and sugarcane, respectively. The incubation time for the transfection process influenced the uptake of vectors by protoplasts. The transfection was highly efficient (68%) at a 30 min incubation period, but longer incubation time decreased transfection rate (Figure 2C). PCR amplification of the pCAMBIA1303 transfected protoplasts from lanes 1–4 clearly indicated the presence of the hptII and gusA genes at 407 bp and 515 bp, respectively (Figure 2E,F).

## 4. Materials and Methods

### 4.1. Optimization of Factors Influencing Protoplast Yield

Healthy leaves (first 3–4 fully developed leaves) were collected from in vitro-grown, 4-week-old *B. rapa* cv. Kenshin plants [1]. Small leaf pieces (approximately 1 g) were sliced into thin strips (approximately 0.5 mm) in a 50-mL Falcon tube containing an enzyme mixture of 40 mL (Sigma-Aldrich, St. Louis, MO, USA) comprising cellulase (1%), macerozyme (0.25%), and pectinase (0.25%), 15 mM CaCl_2_, 25 mM KCl, 0.1% BSA, and 20 mM MES buffer, at pH 5.7 with different concentrations of mannitol (0.2, 0.3, 0.4, 0.5 and 0.6 M). The mixture was placed in a shaking incubator set at 50 rpm for 3 h at 25 ± 2 °C in darkness. After optimizing the mannitol concentration (0.5 M), various cellulase concentrations (0.5, 1, 1.5 and 2%) were tested. All other components were unchanged. After optimizing the cellulase concentrations (1.5%), various incubation periods were studied (1, 2, 3, 4 and 5 h) to optimize incubation time for cell wall digestion.

After completing the enzymatic digestion under optimal conditions, the mixture was gently swirled to release the protoplasts into the suspension, which was then filtered through a 40 µm nylon mesh. The filtrate (~40 mL) was collected in a new Falcon tube, which was subsequently centrifuged at 100× *g* for 3 min. Ten milliliter of chilled W5 solution (175 mM NaCl, 130 mM CaCl_2_, 5 mM KCl, 8 mM glucose, and 3 mM MES, pH 5.7) was gently added along the inner wall of the tube. This step was repeated twice to ensure the enzyme mixture was completely removed by a centrifugation (at 100× *g* for 3 min). The purified protoplast layer (i.e., middle layer) was obtained using a 21% sucrose solution followed by a centrifugation (at 100× *g* for 3 min). Chilled W5 solution was gently added to the recovered protoplast layer and the resulting solution was incubated for 1 h at 4 °C. The protoplast yield was calculated based on Adedeji et al. [20].

### 4.2. Analysis of Protoplast Viability

Protoplast viability was evaluated using fluorescein diacetate (Sigma-Aldrich). Briefly, 1 µL 0.05% fluorescein diacetate was added to a 100 µL aliquot of protoplast solution, which was then incubated for 30 min at room temperature. The protoplasts were examined under ultraviolet light using a fluorescence microscope to assess viability (%) according to the following equation: the number of fluorescent protoplasts/the number of total protoplasts × 100 [20].

### 4.3. Optimization of Factors Influencing Protoplast Transfection with a Binary Vector

The binary vector pCAMBIA1303 (approximately 12 kb) carries *hygromycin phosphotransferase (hptII)* as a selection marker, and *β-glucuronidase A*:*green fluorescent protein 5* (*gusA-mgfp5*) as a fusion gene scorable marker, under the control of the CaMV35S promoter. Different concentrations (10–50 µg) of the pCAMBIA1303 binary vector were used for the transfection of 2 × 10^6^ protoplasts in 100 µL MMG buffer (0.5 M mannitol, 5 mM MES, and 1.5 mM MgCl_2_). Next, 120 µL of 40% PEG solution was added. The resulting solution comprising the protoplasts, vector, and PEG was gently mixed by inverting the tube, which was then incubated for 30 min at room temperature. After optimizing the vector concentration (40 µg), various PEG concentrations (10–50%) were tested. The other components were unchanged. After optimizing the PEG concentration (40%), the incubation period was varied (10–40 min) to determine the optimal incubation time for transfection.

After completing the transfection under optimal conditions, the transfected protoplast mixture was washed three times with 10 mL chilled W5 solution by centrifuging at 100× *g* for 5 min. The WI medium (4 mM MES, 0.5 M mannitol, and 25 mM KCl, pH 5.7) was then slowly added to the pelleted transfected protoplasts, which were then incubated for 48 h at 25 °C in darkness. The control was maintained without the plasmid. The GFP signal in the transfected protoplasts was detected under ultraviolet light using a fluorescence microscope. The transfection efficiency was determined as the number of fluorescent protoplasts/the number of total protoplasts × 100 [20]. 

### 4.4. Confirmation of Vector Integration by PCR

After 48 h incubation under total darkness, the protoplast cells were washed with W5 solution for several times in order to remove non-transfected vectors in the culture. Total genomic DNA was isolated from the incubated protoplast and control (without pCAMBIA1303 transfection) according to manufacturer instructions (GenElute^TM^ Plant genomic DNA miniprep kit; Sigma-Aldrich, Burlington, VT, USA).The presence of *hptII* and *gusA* in the transfected protoplasts was confirmed by a PCR analysis using gene-specific primers, the HiPi Master Premix (Elpis Biotech, Daejeon, Korea), and the following program: 94 °C for 5 min; 30 cycles of 94 °C for 1 min, 55 °C for 1 min, and 72 °C for 1 min; 72 °C for 7 min. The forward and reverse primers for amplifying *gusA* (515 bp) were, respectively, FP (5′-ATTGATCAGCGTTGGTGG-3′) and RP (5′-ACGCGTGGTTACAGTCTTGC-3′), whereas the forward and reverse primers for amplifying *hptII* (407 bp) were, respectively, FP (5′-GATGTTGGCGACCTCGTATT-3′) and RP (5′-GTGTCACGTTGCAAGACCTG-3′). The PCR products were analyzed by 1% (*w/v*) agarose gel electrophoresis and ethidium bromide staining. 

### 4.5. Statistical Analysis

The experiments were repeated three times with three replicates. Data are presented as mean ± SE. The mean separations were performed using Duncan’s multiple range test, and significance was determined at the 5% level (SPSS 17.5; IBM, Armonk, NY, USA).

## 5. Conclusions

In this study, we standardized an effective method for isolating Chinese cabbage protoplasts from leaf mesophylls and subsequently transfecting them with a relatively large binary vector by optimizing relevant critical parameters. To the best of our knowledge, the experiments described herein are the first to optimize specific factors to maximize Chinese cabbage protoplast yield (mannitol and cellulase concentrations, and incubation time), viability and transfection methods (binary vector and PEG concentrations and incubation time). The protocols developed in this study are potentially relevant for future studies related to genome editing, subcellular localizations, gene and protein functions, signal transduction pathways and defense mechanisms.

## Data Availability

The relevant datasets supporting the results of this article are included within the article.
